# Temporal dynamics of positive emotion regulation: insights from facial electromyography

**DOI:** 10.3389/fnhum.2024.1387634

**Published:** 2024-05-15

**Authors:** Sylvia D. Kreibig, James J. Gross

**Affiliations:** Stanford Psychophysiology Laboratory, Department of Psychology, Stanford University, Stanford, CA, United States

**Keywords:** emotion regulation (reappraisal), temporal dynamics analysis, positive affect (PA), negative affect (NA), electromyography (EMG), corrugator supercilii (CS), zygomaticus major (ZM), time course analysis

## Abstract

**Introduction:**

Emotion regulation (ER) is a complex process that manifests gradually over time. This study investigated the temporal dynamics of ER in modifying positive emotions in terms of both negative affect (NA) and positive affect (PA) dimensions.

**Methods:**

After participants had been exposed to pleasant pictures for 8,000 ms, they received instructions to either continue viewing the picture (no regulation) or reappraise it with a neutral meaning (neutralize goal) or negative meaning (transform goal) for another 8,000 ms. We obtained corrugator supercilii and zygomaticus major electromyography (EMG) as objective measures of NA and PA.

**Results:**

For the no-regulation condition, upon instruction onset, we observed maintained low levels of corrugator and high levels of zygomaticus EMG reactivity, indicating sustained PA activation. Compared to the no-regulation condition, for the neutralize goal, we observed no change in corrugator reactivity, which remained at a low level, while zygomaticus reduction started at 1,000 ms after instruction onset, indicating decreased PA and generation of a neutral emotional state. For the transform goal, we observed corrugator increase and zygomaticus decrease both starting at 1,500 ms after instruction onset and co-existing throughout the regulation period. These results indicate increased NA and decreased PA, relating to generation of a negative emotional state. The transform goal differed from the neutralize goal in terms of corrugator increase starting at 2,500 ms after instruction onset. Albeit simultaneous onset of changes on corrugator and zygomaticus reactivity under the transform goal, model-fitting analyses indicated that the best-fitting trajectory was one that first emphasized PA reduction until, at 3,000 ms, it turned into primary NA increase.

**Discussion:**

These distinct temporal patterns highlight the possibility of effecting one-dimensional PA change with the neutralize goal and sequential two-dimensional change (first decreasing PA, then increasing NA) with the transform goal. This research sheds light on the time course of emotional change brought about by different regulatory goals.

## 1 Introduction

The exploration of time-dependent changes has gained increased importance in understanding emotion and its regulation. Although substantial progress has been made, there persists a crucial gap in our knowledge of how emotion regulation (ER) impacts negative affect (NA) and positive affect (PA) response systems over time.

**Figure 1:**
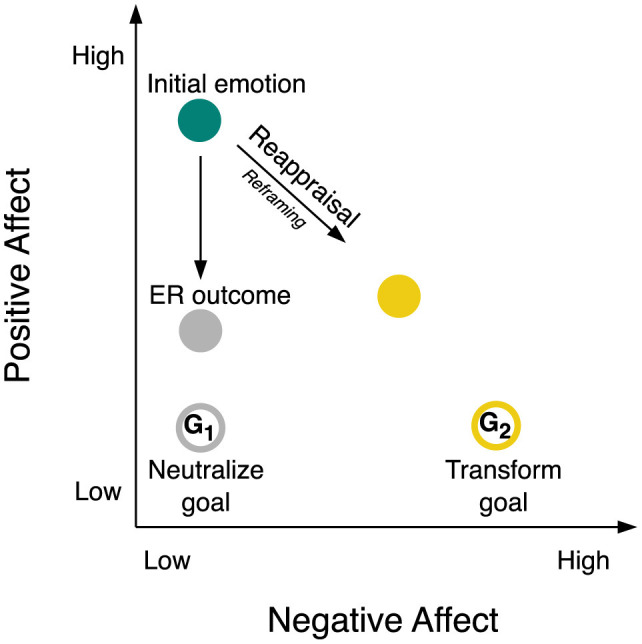
Emotion regulation process components within two-dimensional space of negative and positive affect. The figure illustrates regulation of a highly positive initial emotion (like joy or desire, teal circle) through reappraisal as the ER strategy implemented as reframing as the ER tactic. There may be two different ER outcomes: a less positive and more neutral ER outcome (e.g., neutral emotion, gray circle) may be achieved by pursuing a neutralize goal (*G*_1_; open gray circle); a less positive and more negative ER outcome (like fear or disgust, yellow circle) may be achieved by pursuing a transform goal (*G*_2_; open yellow circle). See sections 1 Introduction and 1.1 Emotion regulation for a detailed explanation of this figure. ER, emotion regulation; *G*_1_, ER goal 1; *G*_2_, ER goal 2.

Emotions unfold dynamically over time (Scherer, 2009a,b; Kuppens and Verduyn, 2017). They undergo constant fluctuations in intensity and quality. Emotions arise from appraisals (Scherer, 2001) and vary on such fundamental dimensions like valence and arousal (Russell, 1980). They manifest in prototypical forms like joy, desire, fear, or disgust (Ekman, 1999), sometimes even blending into mixed emotions (Larsen and McGraw, 2014). One contemporary theory, among others, proposes that emotions occur within an affective space spanned by NA and PA (Cacioppo and Berntson, 1999, a 45°-rotation of the valence–arousal model; Russell and Feldman Barrett, 1999). As shown in Figure 1, the NA dimension spans from low NA to high NA on the *x*-axis. The PA dimension spans from low PA to high PA on the *y*-axis. In this two-dimensional framework, negative emotion, like fear or disgust, occupies the lower right quadrant, neutral emotion resides in the lower left quadrant, positive emotion, like joy or desire, is situated in the upper left quadrant, and mixed emotion is located in the upper right quadrant. We here focus on the down-regulation of positive emotion, characterized by low NA and high PA, represented by the teal circle in Figure 1. While positive emotions generally benefit well-being, down-regulating them can be adaptive at times, such as when navigating social contexts or adhering to long-term goals. For example, to promote goal-directed behavior, we may choose to turn intense joy into a neutral state, e.g., maintaining professionalism during negotiations, or even into a mild aversion, e.g., viewing tempting food with slight disgust.

### 1.1 Emotion regulation

ER empowers individuals to sculpt their emotional experience, shaping what they feel, when they feel it, and how they show it (Gross, 1998). ER actively manages both NA and PA. At its core, ER consists of several parts that interact with each other in a multi-stage process (Gross, 1998, 2015). Regulation begins with identifying the *initial emotion* and adopting an ER goal. Recognizing the need to adjust (or maintain) the initial emotion sparks the *ER goal*. An ER goal frequently targets a different emotional state than the initial emotion (Mauss and Tamir, 2014; Kreibig et al., 2023). Regulation goals may aim to alter the initial emotion's intensity, such as reducing positive emotion to a neutral emotional state as in the above negotiation example, see *G*_1_ in Figure 1. We use the term *neutralize goal* to denote a quantitative ER goal aiming to reduce emotion intensity (Kreibig et al., 2023). A neutralize goal in regulating a positive emotion shifts emotional reactivity toward the lower left quadrant in affective space. Alternatively, goals can change emotion type or quality, e.g., positive to negative as in the above food example, see Figure 1 *G*_2_. We use the term *transform goal* to denote a qualitative ER goal aiming to change emotion type (Kreibig et al., 2023). A transform goal in regulating a positive emotion shifts emotional reactivity toward the lower right quadrant in affective space^1^.

Regulation strategies bridge the gap between initial and target emotion. Cognitive reappraisal, a particularly effective and adaptive ER strategy, modifies the emotional impact of a stimulus by reinterpreting its meaning (Augustine and Hemenover, 2009; Webb et al., 2012). Translating broader ER strategies into concrete actions involves the use of ER tactics, such as reframing, a specific tactic within cognitive reappraisal (McRae et al., 2012; Uusberg et al., 2019). The ER strategy and its implementation through the ER tactic are symbolized with the arrow connecting initial emotion and ER outcome in Figure 1.

The ER outcome or regulated emotion (gray circle in Figure 1) indicates the point in affective space reached after using an ER tactic for a certain duration of time, e.g., seconds or minutes. ER outcomes can deviate from goals due to overshooting (too strong effect), undershooting (weaker effect; Kreibig et al., 2023), or delayed effects. The open and filled gray circles in Figure 1 depict this potential deviation between the target emotion (goal) and the actual outcome after regulation.

### 1.2 Temporal dynamics of emotion regulation

Considering the gradual nature of emotional change, we ask how ER unfolds its influence on emotional outcomes and how the emotional journey progresses within affective space at each point in time. Figure 1 could depict this evolving ER process through a series of gray (and yellow) circles, representing snapshots of the regulated emotion at different points in time, like frames in a movie.

Existing ER frameworks already encompass the concept of temporal dynamics. Amongst other theories, the process model of ER (Gross, 1998, 2015) recognizes that ER effects do not happen instantly but unfold and manifest gradually over time. The model highlights a repeated loop of implementing ER strategies through tactics and continually evaluating their effectiveness, allowing adjustments to maintain, stop, or redirect regulation efforts. How might this process look like in reality?

Imagine a person experiencing an intense positive emotion, like joy or desire (low NA, high PA), at time *t*_0_. They decide to regulate their emotional response by cognitively reappraising the pleasant stimulus, e.g., tempting food, by aiming to either neutralize or transform it. Within, the following process unfolds. As the brain starts reinterpreting the situation, it takes some time until the stimulus's emotional significance has been modified. If reappraisal is successful, central activation of the PA system gradually decreases (*t*_1*A*_ onwards; Krompinger et al., 2008; Schönfelder et al., 2014; Langeslag and Sanchez, 2018; Feng et al., 2023). Depending on the goal, central activation of the NA system will either remain low with a neutralize goal or start to increase with a transform goal (*t*_1*B*_, e.g., generating either a neutral or repulsed emotional state; Kreibig et al., 2022, 2023). The peripheral nervous system subsequently reflects this changed central activation, leading to differences from the unregulated response, in terms of PA at *t*_2*A*_ and NA at *t*_2*B*_ (Schönfelder et al., 2014; Li et al., 2018). Peak reappraisal effects manifest as most significant PA reduction at *t*_3*A*_ and potentially highest NA at *t*_3*B*_. Reappraisal transitions from implementation to monitoring as the desired emotional state is reached^2^, and regulation efforts stabilize. PA settles at a lower level than the initial emotion at *t*_4*A*_, while NA either remains low or might be elevated, depending on the ER goal, at *t*_4*B*_. The peripheral response reflects this central nervous system state at *t*_5*A*_ and *t*_5*B*_, respectively^3^.

Examining this idealized sequence, we see three crucial aspects of the temporal dynamics of ER. Firstly, there is a cascade of events as ER effects develop, highlighting the gradual nature of ER. Secondly, there is a clear distinction between the regulated and unregulated emotional responses. Thirdly, even within ER, the specific timeline depends on the chosen goal. Neutralize goals might be limited to reducing PA, whereas transform goals may not only reduce PA but also increase NA to shift emotional type.

**Figure 2:**
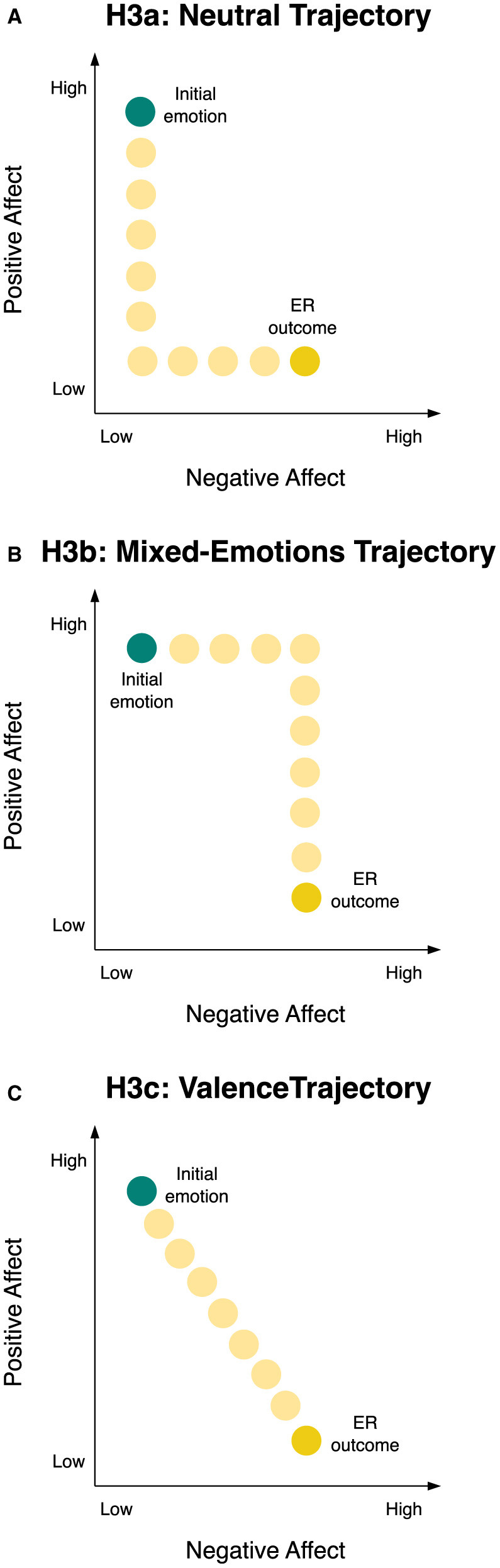
Illustration of Hypotheses 3a, 3b, and 3c for the temporal change trajectory of the transform goal through two-dimensional affective space. Light yellow circles visualize the hypothesized trajectory converting the initial emotion (teal circle) into the ER outcome (yellow circle). **(A)** Illustration of H3a, the neutral trajectory, i.e., sequential change in form of, first, PA reduction and, second, NA increase over time, describing an L-shape trajectory of passing through a neutral emotional state. **(B)** Illustration of H3b, the mixed-emotions trajectory, i.e., sequential change in form of, first, NA increase and, second, PA reduction over time, describing a trajectory of an inverse-step function, passing through a mixed-emotional state. **(C)** Illustration of H3c, the valence trajectory, i.e., concurrent change on NA and PA over time, describing a diagonal change trajectory that corresponds to the valence dimension in affective space, i.e., a 45°-rotation of NA and PA axes. ER, emotion regulation; H3a, Hypothesis 3a; H3b, Hypothesis 3b; H3c, Hypothesis 3c.

The possible temporal sequences for the transform goal include: Sequential change, prioritizing PA reduction followed by NA increase (Pattern A) or vice versa (Pattern B), implying distinct phases changing one affect dimension at a time; or concurrent change, where both PA and NA adjust simultaneously in an intertwined fashion (Pattern C). Pattern A involves reaching a neutral state first (low NA, low PA) before moving onto a negative state (cf. Figure 2A). Pattern B implies intermediate generation of mixed emotions, given the increase in NA before the reduction of PA (cf. Figure 2B). Pattern C navigates affective space by small consecutive increases in NA and decreases in PA over time, tracing a downward diagonal line corresponding to the valence dimension from positive to negative affect (cf. Figure 2C). We next turn to reviewing empirical findings to see what is known about the sequencing of ER effects on NA and PA systems.

### 1.3 Empirical findings

Recent years have seen a surge in research exploring the timing of emotions and their regulation. A key factor behind this progress is the use of psychophysiological methods, which measure how emotions manifest in our bodies. Techniques like facial electromyography (EMG) offer insights with temporal resolution at the sub-second level, among other processes, into the fleeting nature of facial emotional micro-expressions (Tassinary and Cacioppo, 1992; Ekman, 2009; Kim et al., 2022). This allows us to track the moment-by-moment changes during ER, enabling detailed analysis of the timing of these processes.

#### 1.3.1 Unregulated emotional response

For the unregulated emotional response without the explicit engagement of ER, studies show clear patterns in facial muscle reactivity of corrugator supercilii and zygomaticus major muscles for both positive and negative emotions. These reactions happen quickly (milliseconds/seconds) and can last for several minutes. Pleasant stimuli, like images of tempting food, compared to neutral ones, typically trigger zygomaticus activation (smiling) while corrugator (frowning) activity stays flat or slightly reduces. Conversely, unpleasant stimuli, like images of spoiled food, see the opposite pattern with corrugator activation and zygomaticus deactivation.

In particular, corrugator and zygomaticus muscles respond within a few hundred milliseconds to the presentation of emotional stimuli (e.g., faces or scenes; Dimberg and Thunberg, 1998; Neta et al., 2009; Soussignan et al., 2013; Slessor et al., 2014; Mavratzakis et al., 2016) and maintain activation during the first 1,000 to 2,500 ms. Both muscles maintain activity even beyond the initial response, showing continued affective modulation for 4,000 to 8,000 ms after stimulus onset (Tan et al., 2012; Fiacconi and Owen, 2015; 't Hart et al., 2018; Korb et al., 2019; Fuentes-Sánchez et al., 2021). Even after an unpleasant trigger disappears, corrugator reactivity remains elevated, with studies observing sustained activity throughout 8,000 ms of inter-trial interval (Heller et al., 2011; Van Reekum et al., 2011; Heller et al., 2014; Pedersen et al., 2022). In prolonged affective stimulation of 20 to 40 s, e.g., rapid serial visual stimulation (Smith et al., 2006), film clips (van Boxtel, 2010), or stories ('t Hart et al., 2018), both corrugator and zygomaticus continue to show affective differentiation and also respond dynamically to new emotional events, as seen in studies using stories (e.g., 't Hart et al., 2018). Facial expressions maintain their responsiveness to emotions even past one minute of stimulus exposure. Studies using movie clips or blocked static images over 2 to 6 min show ongoing affective differentiation of corrugator and zygomaticus reactivity (Bradley et al., 1996; Codispoti et al., 2008; Golland et al., 2018; Sato et al., 2020). These studies highlight that, without explicit ER, facial responses to emotional stimuli (past or present) persist and evolve over time, with sustained or increased muscle reactivity reflecting ongoing emotional impact.

#### 1.3.2 Regulated emotional response

For down-regulating emotional responses to pleasant stimuli through cognitive reappraisal, we list studies that examined either the time course or time averages of corrugator and zygomaticus reactivity in Table 1. We are aware of only three prior studies that delve into the temporal dynamics (Wu et al., 2012; Schönfelder et al., 2014; Li et al., 2018), while the other six present time-averaged results. Closer inspection of Table 1 reveals that prior studies report a variable range of small to large effect sizes for zygomaticus reduction and small to medium effect sizes for corrugator reactivity, relating to either less decreased or even increased reactivity, compared to the unregulated emotional response within the first few one hundred to one thousand milliseconds that is sustained for several seconds—and possibly minutes—thereafter (Table 1).

**Table 1:** Overview of studies on the temporal dynamics of down-regulating emotional responses to pleasant stimuli through cognitive reappraisal using facial electromyography or observer coding as measures of corrugator supercilii and zygomaticus major muscle activity.

**Study**	**Reappraisal tactic**	**Measure**	**Effect timing (ms)**	**Effect size**	**Effect (statistical analysis)**
*Pre-stimulus delivery of cognitive reappraisal instructions*					
Wu et al. (2012)	Appraisal frames	↔CS^†^	*n.s*.	Medium	stimulus valence × appraisal frame^1^
		↓ZM^†^	500–3,000	Medium	Pleasant–positive vs. pleasant–neutral
Schönfelder et al. (2014)	Detached/distancing	↓ZM^†^	2,000–5,000	Large	ER strategy × time^2^
Baur et al. (2015)	Unspecified	↔CS^†^	*n.s*.	NA	Down-regulation vs. no regulation
		↓ZM^†^	4-s avg.	Small	Down-regulation vs. no regulation
Bernat et al. (2011)	Detached/distancing	↑CS^†^	6-s avg.	Medium	Stimulus valence × CR direction^3^
		↓ZM^†^	6-s avg.	Medium	Stimulus valence × CR direction^3^
Kreibig et al. (2022)	Situation-focused	↑CS^†^	~25-s avg.	Medium	Negative vs. no ER goal^4^
		↓ZM^†^	~25-s avg.	Large	Negative vs. no ER goal^4^
Lalot et al. (2014)	Detached/distancing	↓ZM^‡^	~164-s avg.	Small	ER strategies contrast^5^
Gruber et al. (2014)	Detached/distancing	↓ZM^‡^	~166-s avg.	Large	CR vs. no regulation^6^
***Post-stimulus delivery of cognitive reappraisal instructions***					
Kreibig et al. (2023)	Situation-focused	↔CS^†^	*n.s*.	Small	Quantitative ER goal vs. no regulation
		↓ZM^†^	8-s avg.	Medium	Quantitative ER goal vs. no regulation
		↑CS^†^	8-s avg.	Medium	Qualitative ER goal vs. no regulation
		↓ZM^†^	8-s avg.	Small	Qualitative ER goal vs. no regulation
Li et al. (2018)	Situation-focused	↔ZM^†^	*n.s*.	Small	Decrease vs. no regulation

We categorized effect sizes as small, medium, or large according to Cohen (1988). ^†^ Electromyography (EMG) for quantification of muscle activity. ^‡^Facial Action Coding System (FACS; Ekman and Friesen, 1978) for quantification of muscle activity; Gruber et al. (2014) coded AU12 + AU6 (lip corner puller + cheek raiser); Lalot et al. (2014) coded AU12 (lip corner puller). We caution that EMG responses may be stronger in magnitude than FACS responses (Tassinary and Cacioppo, 1992). ^1^unpleasant, pleasant pictures × neutral frame, stimulus valence-consistent frame. ^2^CR, distraction × seconds 1, 2, 3, 4, 5. ^3^stimulus valence × enhance, suppress. ^4^negative ER goal = emphasis on the repulsive, painful, or negative elements of the situation. ^5^suppression vs. (CR, mindfulness) vs. no regulation. ^6^CR vs. uninstructed condition for the happy film clip across bipolar and healthy control participants (*n.s*. group difference). avg., average; CR, cognitive reappraisal; CS, corrugator supercilii; ER, emotion regulation; ZM, zygomaticus major.

As we summarize in the upper section of Table 1, seven of the nine studies utilized pre-stimulus (anticipatory) ER paradigms, where reappraisal instructions appear before the elicitation of emotion (e.g., Urry, 2009). Two studies out of these reported time course analyses. One study demonstrated that when presenting appraisal frames, i.e., 2,000 to 4,000 ms long auditory narratives preceding pleasant pictures, zygomaticus reactivity was lower in the neutral than positive framing condition from 500 to 3,000 ms of the 4,000 ms pleasant picture presentation while corrugator reactivity did not differ significantly (suggestive of a neutralize goal; Wu et al., 2012). The other study showed that detached reappraisal reduced zygomaticus reactivity compared to unregulated viewing within 2,000 ms, and this difference was sustained throughout the remainder of the 5,000 ms pleasant picture presentation (Schönfelder et al., 2014).

Five studies reported analysis of time-averaged results. Reappraising pleasant pictures for 4 to 6 s reduced zygomaticus reactivity and either left corrugator reactivity at baseline levels (rather than decreasing it in response to the pleasant stimuli as in the study's no-regulation condition, suggestive of a neutralize goal; Baur et al., 2015) or even increased corrugator reactivity (suggestive of increased negative emotion as under a transform goal; Bernat et al., 2011). Similarly, situation-focused reappraisal with an explicit transform ER goal, i.e., emphasizing the repulsive, painful, or negative elements, of brief (20–30 s) amusing film clips reduced zygomaticus reactivity and increased corrugator reactivity (Kreibig et al., 2022). Detached reappraisal of film clips of several minutes duration targeted at inducing amusement, happiness, or tenderness reduced zygomaticus reactivity quantified in terms of AU12 (Lalot et al., 2014) or AU6+AU12 (Gruber et al., 2014), respectively (cf. Facial Action Coding System; Ekman and Friesen, 1978).

Only two studies utilized a post-stimulus (online) ER paradigm, where reappraisal instructions appear after initial emotion elicitation (e.g., Urry, 2009), as we summarize in the lower section of Table 1. Li et al. (2018, study 2) reported no effect on zygomaticus reactivity of situation-focused reappraisal instructions at 4,000 ms after picture onset over the ensuing 12,000 ms regulation period compared to the unregulated emotional response. In contrast, we (Kreibig et al., 2023), reporting time-averaged results, found differentiated effects on corrugator and zygomaticus responses of neutralize and transform goals in reappraising pleasant pictures over 8,000 ms after a preceding 8,000 ms free-viewing. The neutralize goal reduced zygomaticus reactivity with no change in corrugator reactivity but the transform goal reduced zygomaticus reactivity and increased corrugator reactivity. These results suggest that the two ER goals, which both decreased positive emotion, generated distinct ER outcomes. However, the temporal unfolding of these ER goal effects remains unclear.

### 1.4 The present study

The present study aimed to fill this crucial gap in our current knowledge by empirically studying how neutralize and transform ER goals influence the temporal dynamics of reappraising pleasant stimuli where a positive emotion has already been elicited. Our focus was on testing whether the transition from the initial emotion to the regulated emotion occurred sequentially or concurrently over time on NA and PA response systems within a two-dimensional affective space. By holding constant key variables—initial emotion (PA), strategy (cognitive reappraisal), and tactic (changing the cognitive representation of current circumstances)—we were able to single out the impact of the neutralize vs. the transform goal on emotional responses over time, comparing them to unregulated emotional responses and each other. We investigated the following hypotheses:

#### 1.4.1 Hypothesis 1

We hypothesized that the unregulated positive emotional response would exhibit response maintenance, characterized by a stable level of relatively unchanged low NA and high PA over time.

#### 1.4.2 Hypothesis 2

We hypothesized a uni-dimensional trajectory with no alteration in NA and a gradual reduction in PA over time for the neutralize goal.

#### 1.4.3 Hypothesis 3

Because the analysis of time averages indicated change on both the NA and PA dimensions for the transform goal compared to the unregulated emotional response (Kreibig et al., 2023), we postulated three potential temporal sequences for the effects of the transform goal, depicted in Figure 2:

The first scenario was sequential change of first a reduction in PA and then an increase in NA over time. This corresponds to an L-shaped trajectory transitioning through a neutral emotional state, illustrated in Figure 2A and henceforth referred to as the *neutral trajectory* (H3a).

The second scenario was sequential change of first an increase in NA and then a reduction in PA over time. This corresponds to a trajectory resembling an inverse-step function, transitioning through a mixed-emotional state, illustrated in Figure 2B and henceforth referred to as the *mixed-emotions trajectory* (H3b).

The third scenario was concurrent change on NA and PA over time, depicting a downward diagonal trajectory in affective space. This trajectory aligns with the valence dimension, i.e., a 45°-rotation of NA and PA axes, as depicted in Figure 2C and henceforth referred to as the *valence trajectory* (H3c).

## 2 Materials and methods

The current dataset was derived from a study originally reported in Kreibig et al. (2023). Whereas the initial paper addressed the findings from the analysis of time averages, the current paper reports the outcomes of time course analyses.

### 2.1 Participants

Participants for this study were chosen via phone screening. Individuals proficient in English, aged between 18 and 30 years, and possessing a minimum of eight years of education were enrolled. Exclusion criteria included individuals with a history of smoking or those who had conditions or were taking medications that could affect the cardiovascular, respiratory, autonomic, or central nervous systems. The study also excluded individuals with past psychiatric diagnoses (see Kreibig et al., 2023, for details). Participants were recruited through Stanford University, community participant pools, online advertisements, and local flyers. Participants voluntarily enrolled in this study, which was promoted as exploring the psychophysiological impact of processing affective images.

Analysis included 156 participants (83 females, 72 males, 1 other) who completed the study. The average age was 20.82 (*SD* = 2.94) years, with 9.0% identifying as African American, 21.8% as Asian American, 50.0% as Caucasian, 10.3% as Hispanic, 2.6% as Native American, 5.1% as other, and 1.3% declining to state. Participants were compensated with either course credit or $70.

### 2.2 Materials

We used 105 color photographs from the International Affective Picture System (Lang et al., 2008, *N* = 65) and an in-house affective picture library (Kreibig et al., 2023, *N* = 40). Among these images, 45 had unpleasant valence (*M* = 2.33, *SD* = 0.29, range: 1.80–3.14), 45 had pleasant valence (*M* = 7.60, *SD* = 0.28, range: 6.76–8.34), and 15 had neutral valence (*M* = 5.02, *SD* = 0.12, range: 4.84–5.27). Image selection relied on combined emotional ratings by both female and male participants, utilizing a 9-point scale with the midpoint at 5. This report examines the temporal dynamics of emotion and ER in response to pleasant pictures; responses to unpleasant pictures are the topic of another report (Kreibig and Gross, 2024).

### 2.3 Apparatus

Stimuli were presented on a 19-inch computer monitor situated 55 cm away from participants in low ambient light conditions, using Inquisit 3.0.6.0 (Millisecond Software, Seattle, WA). Participants entered responses using a keyboard, and their compliance was monitored via a concealed camera. Physiological data were recorded using a BioNex 8-slot chassis (Mindware Technologies, Grahanna, OH) equipped with two 4-channel biopotential amplifiers (Model 50-371102-00) and transmitted to a computer for storage and monitoring using BioLab 2.4 with 16-bit resolution (Mindware Technologies, Grahanna, OH).

### 2.4 Measures

#### 2.4.1 Emotional expression

Surface EMG (μV) measured emotional expression on the left corrugator supercilii and zygomaticus major muscles at a sampling rate of 1,000 Hz, adhering to established protocols (Fridlund and Cacioppo, 1986). Miniature (4 mm) Beckman Ag/AgCl electrodes filled with Teca gel (Oxford Instruments) served for measurement. Skin prep involved cleaning and abrasion until impedance between electrodes was below 10 kΩ for optimal signal quality. Recording sites had 1 cm interelectrode distance (center-to-center). A hardware 500-Hz anti-aliasing low-pass filter was applied before digitizing the EMG-signal. Signals were preprocessed with 60-Hz notch filter, 20–500 Hz digital bandpass filter, and rectification and 10-ms running average smoothing for analysis.

### 2.5 Procedure

The data were collected as part of a multi-session study, which included the following sessions: online survey (1 h), behavioral session (1.5 h), experimental session (3 h).

This manuscript focuses on the experimental session's psychophysiological assessment of a reappraisal task. With participants' informed consent and unaware of study goals, they were seated before a computer screen with physiological sensors attached. Participants reviewed task instructions and practiced the task they were previously familiarized with during the behavioral session using example pictures. The experiment commenced upon the experimenter's exit.

In each trial of the reappraisal task, participants were instructed to rest (8,000 ms), fixate on a white cross (1,000–3,000 ms), view and understand the content of a picture (8,000 ms), reappraise the picture as it remained on the screen according to ER goal instructions (8,000 ms), and complete three rating scales (each 4,000 ms; not relevant here; for results see Kreibig et al., 2023). ER instructions were displayed beneath the picture and directed participants to either continue viewing (VIEW; no regulation) or to reinterpret the pleasant picture in a neutral way (NEUTRALIZE; neutralize goal) or a negative way (TRANSFORM; transform goal).

The reappraisal task followed a fully within-participant design, consisting of 15 trials for each combination of emotional stimuli and ER instructions (for details, see Kreibig et al., 2023). The presentation order of pictures, ER instructions, and rating scales was randomized across trials. Following a manipulation check, participants were disconnected from the physiological equipment, debriefed, compensated, and thanked. We provide the procedures manual on the study's webpage at osf.io/7c4zj.

### 2.6 Data reduction

EMG data processing utilized biosignal analysis software in R (Kreibig et al., 2013; R Core Team, 2023). Epochs analyzed included a pre-stimulus rest baseline (0–8,000 ms prestimulus onset) and picture viewing with reappraisal (0–8,000 ms post-instruction, capturing the regulated response). Within each epoch, 500-ms averages were computed, with outliers exceeding 2.5 standard deviations from the mean removed and replaced with missing values. Percentage change scores were calculated for each time point within the reappraisal period relative to the average pre-stimulus baseline, resulting in 16 500-ms averages per participant per trial.

### 2.7 Data analysis

Statistical analyses were conducted in R (version 4.3.1; R Core Team, 2023) utilizing the effsize (version 0.8.1; Torchiano, 2020) and psych (version 2.3.12; Revelle, 2023) packages. This study did not utilize preregistered materials or analyses. All research materials, data, and analysis code will be openly accessible at the OSF repository for transparency and reproducibility: https://osf.io/7c4zj.

#### 2.7.1 Preliminary analyses

Initial analyses addressed (a) the stability of baseline measures and (b) the independence of corrugator and zygomaticus responses.

##### 2.7.1.1 Baseline stability

Baseline stability of corrugator and zygomaticus EMG was assessed using Cronbach's α for internal consistency (Cronbach, 1951). Internal consistency was computed individually for each participant across 45 repetitions of 16 time points (720 data points per participant), resulting in 156 individual α scores. The mean α, obtained by applying Fisher's *r*-to-*z* transform and subsequent inverse transformation, represented the overall consistency measure. Values exceeding 0.7 were considered acceptable (Nunnally and Bernstein, 1994).

##### 2.7.1.2 Independence of corrugator and zygomaticus response channels

The independence of corrugator and zygomaticus EMG responses, crucial for the two-dimensional affective space model, was investigated by calculating the correlation between their average percent change scores (one value per participant per muscle). A non-significant correlation coefficient would support the assumption of independence.

#### 2.7.2 Primary analyses

##### 2.7.2.1 Analyses addressing hypothesis 1

Paralleling the baseline stability analysis, Cronbach's α (Cronbach, 1951) assessed the internal consistency of emotional reactivity during the no-regulation condition (no explicit ER goal engagement). The same procedure with 15 repetitions of 8,000-ms no-regulation epochs (16 time points each, totaling 240 data points per participant) yielded 156 individual alpha scores (one per participant).

##### 2.7.2.2 Analyses addressing hypothesis 2

**Figure 3:**
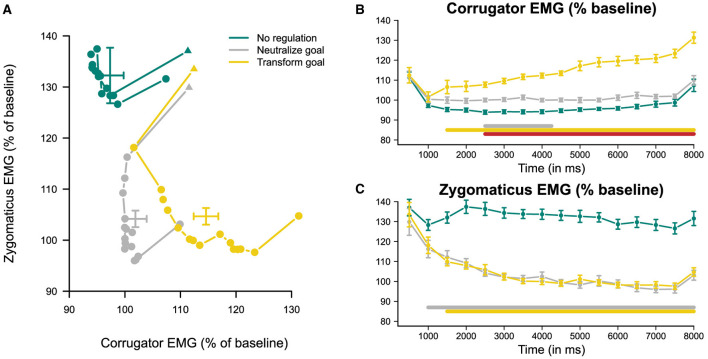
Illustration of the time courses of emotional responses to pleasant stimuli under the no-regulation condition and under cognitive reappraisal with neutralize or transform emotion regulation (ER) goals on corrugator supercilii and zygomaticus major electromyography (EMG) reactivity. **(A)** displays responses within two-dimensional space, constructed with corrugator and zygomaticus as independent dimensions. **(B, C)** display responses within one-dimensional space as plots over time of corrugator and zygomaticus reactivity, respectively. Reactivity was quantified as percent change from each trial's preceding rest baseline. Error bars indicate 95% Cousineau-Morey within-subject confidence intervals (Baguley, 2012). **(A)** Intersections of 95% confidence intervals reflect condition means averaged over time, as originally reported in Kreibig et al. (2023). **(B, C)** The presence of a solid bar above the *x*-axis indicates periods of time in which the ER goal conditions differed. A gray bar reflects differences between neutralize and no-regulation conditions; a yellow bar reflects differences between transform and no-regulation conditions; and a red bar reflects differences between transform and neutralize conditions.

To assess the temporal influence of the neutralize goal on both corrugator and zygomaticus reactivity compared to the no-regulation condition, we employed dependent-samples *t*-tests at each of the 16 time points (500-ms averages each) across the trial duration. Cohen's *d* effect sizes with 95% confidence intervals quantified the magnitude of differences, categorized as small (≥ 0.2), medium (≥ 0.5), or large (≥ 0.8; Cohen, 1988). Mean participant values with 95% Cousineau-Morey within-subject confidence intervals are displayed in the time course plots (Figures 3A–C; Baguley, 2012). To control for multiple comparisons across time points, we used the Bonferroni correction (Dunn, 1961) to adjust the significance level α to 0.003125 (0.05/16).

##### 2.7.2.3 Analyses addressing hypothesis 3

The analysis of the transform goal's influence on corrugator and zygomaticus reactivity as contrasted to the no-regulation condition mirrored the no-regulation vs. neutralize comparison described in Section 2.7.2.2. Furthermore, to evaluate the transform goal's specificity, its effect was directly contrasted with the neutralize goal's impact. This analysis employed the identical set of statistical procedures.

To formally determine which hypothesized response trajectory (Figure 2) best aligns with observed data, we assessed the goodness-of-fit for each. We constructed the following trajectory models: The neutral trajectory (H3a), describing sequential change with PA reduction followed by NA increase, was modeled by a path intersecting the averaged initial emotion and ER outcome of the transform condition in the lower left quadrant. The mixed-emotions trajectory (H3b), describing sequential change with NA increase followed by PA reduction, was modeled by a path intersecting the averaged initial emotion and ER outcome in the upper right quadrant. The valence trajectory (H3c), describing concurrent NA and PA change, was modeled by a one-dimensional diagonal path from initial emotion to ER outcome (shortest distance). Within each hypothesized trajectory model (neutral, mixed-emotions, and valence), points along the path were assumed to be equally spaced, ensuring consistent evaluation across time for model comparison. For each trajectory model, we calculated the point-by-point deviation between the modeled and observed paths using mean squared deviation (*MSD*). The model with the smallest deviation was identified as the best fit.

## 3 Results

### 3.1 Preliminary results

#### 3.1.1 Baseline stability

To test whether assessment of baseline activation of corrugator and zygomaticus EMG was sufficiently stable over the 8,000-ms rest baseline preceding each picture, we calculated Cronbach's α. For corrugator baseline activity, scores ranged from 0.794 to 0.996 across individuals, with an average α of 0.970. For zygomaticus baseline activity, scores ranged from 0.555 to 0.981 across individuals, with an average α of 0.866^4^. For both corrugator and zygomaticus, baseline activity showed satisfactory internal consistency. These results support that the study's repeated rest baseline condition generated a relatively stable state of baseline corrugator and zygomaticus EMG activity.

#### 3.1.2 Independence of corrugator and zygomaticus response channels

To test whether corrugator and zygomaticus reactivity could be considered independent response dimensions, we calculated the correlation between percent change scores of corrugator and zygomaticus EMG reactivity. We found a correlation of *r* = −0.10, *t*(154) = −1.26, *p* = 0.21, 95% CI (−0.25, 0.06). These results support the assumption of uncorrelated response channels.

### 3.2 Primary results

#### 3.2.1 Results for hypothesis 1

As shown in Figures 3A–C, the no-regulation condition, displayed in teal, evidenced little to no appreciable change on both corrugator and zygomaticus reactivity in response to the onset of instructions (at 0 ms) and the subsequent 8,000 ms of trial duration. This interpretation, based on visual inspection, is supported by our analyses of the stability of the emotional response. For corrugator reactivity, scores ranged from 0.410 to 0.998 across individuals, with an average α of 0.982^5^. For zygomaticus reactivity, scores ranged from –0.407 to 0.998 across individuals, with an average α of 0.938^6^. These results indicate fairly stable levels of low corrugator reactivity and heightened zygomaticus reactivity that were maintained throughout the 8,000-ms regulation period. Thus, as we had expected, negative emotional reactivity remained low and positive emotional reactivity remained high under the no-regulation condition.

#### 3.2.2 Results for hypothesis 2

For the neutralize goal, we had expected that there would be no change in corrugator reactivity and a gradual decrease in zygomaticus reactivity over time. We report results of pairwise *t*-tests against the no-regulation condition over time in Tables 2 and 3 for corrugator and zygomaticus reactivity, respectively, and present the corresponding time course plots in gray in Figures 3A–C. We generally found no differences in corrugator reactivity over time between the neutralize and no-regulation conditions, except for a brief small-effect-size increase for 2,000 ms. Corrugator reactivity increased by 6.1% at 2,500 ms after instruction onset for the neutralize compared to the no-regulation condition and continued to be elevated until 4,000 ms, with the largest increase occurring at 3,500 ms (a small-effect-size increase of 7.2%). As hypothesized, a small-effect-size decrease of 12.0% in zygomaticus reactivity first became significant at 1,000 ms after instruction onset and continued to decrease, with the greatest differentiation at 7,000 ms of the 8,000-ms regulation period (a medium-effect-size decrease of 32.3%).

**Table 2:** Pairwise comparisons between emotion regulation goals for corrugator supercilii electromyography reactivity by 500-ms time averages.

**Cond**.	**500**	**1,000**	**1,500**	**2,000**	**2,500**	**3,000**	**3,500**	**4,000**	**4,500**	**5,000**	**5,500**	**6,000**	**6,500**	**7,000**	**7,500**	**8,000**
*No-Regulation*																
*M*	111.3	97.3	95.2	95.3	93.9	94.2	94.1	94.2	94.7	95.2	95.6	95.8	96.7	97.9	98.7	107.4
*SD*	61.3	50.2	51.6	50.6	49.5	49.6	49.1	49.1	49.8	52.6	51.5	56.0	60.1	66.6	69.6	82.7
***Neutralize***																
*M*	111.5	100.4	100.4	99.7	100.0	100.2	101.3	100.2	100.1	100.0	100.0	101.2	102.4	101.7	102.0	109.9
*SD*	60.9	60.1	58.7	51.4	50.1	50.4	56.7	50.4	49.8	51.8	45.8	54.2	58.6	48.9	47.3	49.0
***Transform***																
*M*	112.4	101.6	106.5	106.9	107.7	109.6	111.6	112.3	113.5	117.1	119.0	119.6	120.3	120.8	123.4	131.3
*SD*	55.4	50.4	96.8	78.6	56.9	59.3	68.9	65.8	68.6	88.7	91.5	83.0	77.5	78.2	80.0	79.1
***Neutralize vs. no-regulation***																
*t*	0.12	1.60	2.34	2.70	3.41	3.31	3.63	3.04	2.70	2.33	2.24	2.29	2.36	1.69	1.38	0.94
*p*	0.91	0.11	0.021	0.008	<0.001	0.001	<0.001	0.003	0.008	0.021	0.026	0.023	0.019	0.093	0.17	0.35
	–	–	^*^	^**^	^***^	^**†^	^***^	^**†^	^**^	^*^	^*^	^*^	^**^	.	–	–
*d*	0.01	0.15	0.23	0.24	0.33	0.32	0.37	0.30	0.28	0.24	0.23	0.25	0.25	0.17	0.14	0.08
CI(*d*)	–0.11	–0.03	0.03	0.06	0.13	0.13	0.16	0.10	0.07	0.03	0.03	0.03	0.04	–0.03	–0.06	–0.09
	0.13	0.33	0.42	0.42	0.52	0.52	0.58	0.51	0.49	0.44	0.44	0.46	0.46	0.36	0.34	0.25
***Transform vs. no-regulation***																
*t*	0.73	2.99	3.95	4.53	6.63	6.92	6.74	6.75	7.06	6.44	6.77	6.84	6.96	6.43	6.68	6.17
*p*	0.46	0.003	<0.001	<0.001	<0.001	<0.001	<0.001	<0.001	<0.001	<0.001	<0.001	<0.001	<0.001	<0.001	<0.001	<0.001
	–	^**^	^***^	^***^	^***^	^***^	^***^	^***^	^***^	^***^	^***^	^***^	^***^	^***^	^***^	^***^
*d*	0.04	0.23	0.39	0.45	0.67	0.73	0.75	0.75	0.76	0.72	0.75	0.75	0.77	0.71	0.74	0.64
CI(*d*)	–0.07	0.08	0.19	0.24	0.45	0.49	0.50	0.50	0.52	0.47	0.50	0.50	0.52	0.47	0.49	0.41
	0.14	0.39	0.59	0.66	0.89	0.96	0.99	1.00	1.01	0.97	1.00	0.99	1.02	0.95	0.98	0.86
***Transform vs. neutralize***																
*t*	0.53	0.70	2.28	2.76	3.86	4.30	4.17	5.00	5.21	5.26	5.67	5.58	5.81	5.85	6.37	6.60
*p*	0.60	0.49	0.024	0.007	<0.001	<0.001	<0.001	<0.001	<0.001	<0.001	<0.001	<0.001	<0.001	<0.001	<0.001	<0.001
	–	–	^*^	^**^	^***^	^***^	^***^	^***^	^***^	^***^	^***^	^***^	^***^	^***^	^***^	^***^
*d*	0.03	0.06	0.22	0.27	0.36	0.43	0.42	0.50	0.54	0.55	0.61	0.57	0.59	0.64	0.69	0.64
CI(*d*)	–0.09	–0.11	0.03	0.07	0.17	0.22	0.21	0.29	0.32	0.33	0.38	0.35	0.37	0.40	0.46	0.43
	0.15	0.23	0.41	0.46	0.56	0.63	0.62	0.71	0.76	0.77	0.84	0.79	0.80	0.87	0.93	0.85

Cells marked with yellow background indicate time points that pass the significance level adjusted for multiple comparisons. – *p* > .10, . *p* ≤ .10, ^*^p ≤ .05, ^**^p ≤ .01, ^†^*p* < .003125, the significance level adjusted for multiple comparisons, ^***^p ≤ .001, CI, confidence interval, lower and upper bounds; Cond., condition(s).

**Table 3:** Pairwise comparisons between emotion regulation goals for zygomaticus major electromyography reactivity by 500-ms time averages.

**Cond**	**500**	**1,000**	**1,500**	**2,000**	**2,500**	**3,000**	**3,500**	**4,000**	**4,500**	**5,000**	**5,500**	**6,000**	**6,500**	**7,000**	**7,500**	**8,000**
*No-Regulation*																
*M*	137.1	128.3	132.1	137.5	136.4	134.4	133.8	133.7	133.1	132.7	132.2	128.7	129.7	128.3	126.6	131.6
*SD*	121.2	111.0	116.0	138.4	132.6	124.1	118.8	125.7	121.5	126.7	127.5	125.3	128.4	117.7	111.3	114.1
***Neutralize***																
*M*	129.9	116.3	112.1	109.2	104.2	102.2	101.5	102.5	99.4	98.3	100.3	98.7	96.8	96.0	96.2	103.1
*SD*	122.0	94.3	86.1	69.8	51.2	47.8	47.9	63.4	39.9	41.3	70.6	50.5	42.1	34.2	35.6	41.4
***Transform***																
*M*	133.5	118.2	109.9	108.0	105.9	102.4	100.2	99.9	99.0	101.2	99.5	98.2	98.2	98.2	97.6	104.7
*SD*	129.8	92.9	65.1	59.8	77.9	51.9	40.5	42.4	40.5	56.0	39.8	37.9	41.2	40.5	39.3	42.8
***Neutralize vs. No-Regulation***																
*t*	–1.60	–3.35	–4.56	–5.56	–5.98	–6.63	–6.32	–5.79	–6.82	–6.15	–5.53	–5.49	–6.13	–6.83	–6.78	–6.13
*p*	0.11	0.001	<0.001	<0.001	<0.001	<0.001	<0.001	<0.001	<0.001	<0.001	<0.001	<0.001	<0.001	<0.001	<0.001	<0.001
	–	^**†^	^***^	^***^	^***^	^***^	^***^	^***^	^***^	^***^	^***^	^***^	^***^	^***^	^***^	^***^
*d*	–0.11	–0.21	–0.36	–0.44	–0.52	–0.53	–0.61	–0.57	–0.63	–0.63	–0.61	–0.58	–0.66	–0.71	–0.71	–0.66
CI(*d*)	–0.24	–0.33	–0.52	–0.60	–0.70	–0.70	–0.82	–0.78	–0.83	–0.85	–0.84	–0.81	–0.89	–0.94	–0.95	–0.90
	0.03	–0.09	–0.20	–0.28	–0.34	–0.36	–0.41	–0.36	–0.43	–0.41	–0.37	–0.36	–0.43	–0.48	–0.48	–0.43
***Transform vs. No-Regulation***																
*t*	–0.93	–2.73	–5.13	–5.52	–5.15	–6.36	–7.00	–6.59	–6.89	–5.63	–6.05	–5.92	–6.12	–6.48	–6.77	–6.34
*p*	0.35	0.007	<0.001	<0.001	<0.001	<0.001	<0.001	<0.001	<0.001	<0.001	<0.001	<0.001	<0.001	<0.001	<0.001	<0.001
	–	^**^	^***^	^***^	^***^	^***^	^***^	^***^	^***^	^***^	^***^	^***^	^***^	^***^	^***^	^***^
*d*	–0.06	–0.18	–0.40	–0.47	–0.53	–0.55	–0.58	–0.58	–0.64	–0.57	–0.63	–0.56	–0.61	–0.64	–0.64	–0.58
CI(*d*)	–0.18	–0.31	–0.56	–0.65	–0.74	–0.73	–0.75	–0.77	–0.84	–0.78	–0.85	–0.76	–0.82	–0.86	–0.84	–0.78
	0.06	–0.05	–0.24	–0.30	–0.31	–0.37	–0.40	–0.40	–0.44	–0.35	–0.40	–0.36	–0.39	–0.43	–0.44	–0.39
***Transform vs. Neutralize***																
*t*	0.88	0.71	–0.85	–0.57	0.69	0.13	–0.78	–1.12	–0.28	1.59	–0.38	–0.29	0.76	1.54	0.93	0.94
*p*	0.38	0.48	0.40	0.57	0.49	0.90	0.44	0.27	0.78	0.11	0.70	0.77	0.45	0.13	0.35	0.35
	–	–	–	–	–	–	–	–	–	–	–	–	–	–	–	–
*d*	0.05	0.04	–0.06	–0.05	0.06	0.01	–0.07	–0.11	–0.02	0.14	–0.03	–0.03	0.07	0.14	0.09	0.08
CI(*d*)	–0.07	–0.07	–0.19	–0.20	–0.12	–0.16	–0.26	–0.30	–0.18	–0.03	–0.21	–0.20	–0.12	–0.04	–0.10	–0.09
	0.17	0.14	0.08	0.11	0.25	0.18	0.11	0.08	0.14	0.32	0.14	0.15	0.27	0.33	0.27	0.25

Cells marked with yellow background indicate time points that pass the significance level adjusted for multiple comparisons. – *p* > .05, ^**^ *p* ≤ .01, ^†^*p* < .003125, the significance level adjusted for multiple comparisons, ^***^p ≤ .001, CI, confidence interval, lower and upper bounds; Cond., condition(s).

These results of relatively unchanged, low corrugator reactivity (albeit briefly diverging from the no-regulation condition) and gradually decreasing zygomaticus reactivity over time are in line with the neutralize condition generating a neutral emotional state, resulting in a transition from the upper left to the lower left quadrant of affective space (Figure 3A), as we had predicted.

#### 3.2.3 Results for hypothesis 3

##### 3.2.3.1 Transform vs. no-regulation

For the transform goal, we had hypothesized that the increase in corrugator and decrease in zygomaticus reactivity would happen either sequentially or concurrently. As summarized in Tables 2 and 3 and shown with the corresponding time course plots in yellow in Figures 3A–C, pairwise repeated *t*-tests indicated that a small-effect-size increase of 11.2% in corrugator reactivity for the transform condition in comparison to the no-regulation condition first emerged at 1,500 ms after instruction onset. Corrugator reactivity continued to increase until it reached its greatest difference at 6,500 ms (a medium-effect-size increase of 23.6%) and was sustained throughout the 8,000-ms regulation period. A small-effect-size decrease of 22.2% in zygomaticus reactivity became significant at 1,500 ms after instruction onset and continued to decrease throughout the 8,000-ms regulation period, with the greatest differentiation at 4,500 ms (a medium-effect-size increase of 34.1%). These findings show that the transform goal in moving from a positive toward a negative emotional state increased corrugator reactivity and decreased zygomaticus reactivity, resulting in a transition from the upper left to the lower right quadrant of affective space (Figure 3A). While both responses started simultaneously and co-existed throughout the regulation period, zygomaticus reactivity peaked earlier than corrugator reactivity.

##### 3.2.3.2 Transform vs. neutralize (ER specificity)

The comparison between the two ER goals addressed which change was specific to the transform goal compared to the neutralize goal. Drawing on results reported in Tables 2 and 3 and time courses depicted in gray and yellow in Figures 3A–C, a small-effect-size increase of 7.7% in corrugator reactivity first became significant at 2,500 ms after instruction onset for the transform compared to the neutralize goal (cf. Figure 3B). This increase continued for the remainder of the 8,000-ms regulation period with the largest differentiation occurring at 7,500 ms (a medium-effect-size increase of 21.4%). As expected, we found that there were no significant differences in zygomaticus reactivity between the two regulation goals (cf. Figure 3C), indicating that they shared a similar trajectory of zygomaticus reduction. These results indicate additional corrugator activation by the transform goal. Results demonstrate that both regulation goals shared, at first, the decrease in zygomaticus reactivity. The transform goal started diverging from this shared trajectory with the neutralize goal at 2,500 ms after instruction onset with an increase in corrugator reactivity.

##### 3.2.3.3 Model fitting

**Figure 4:**
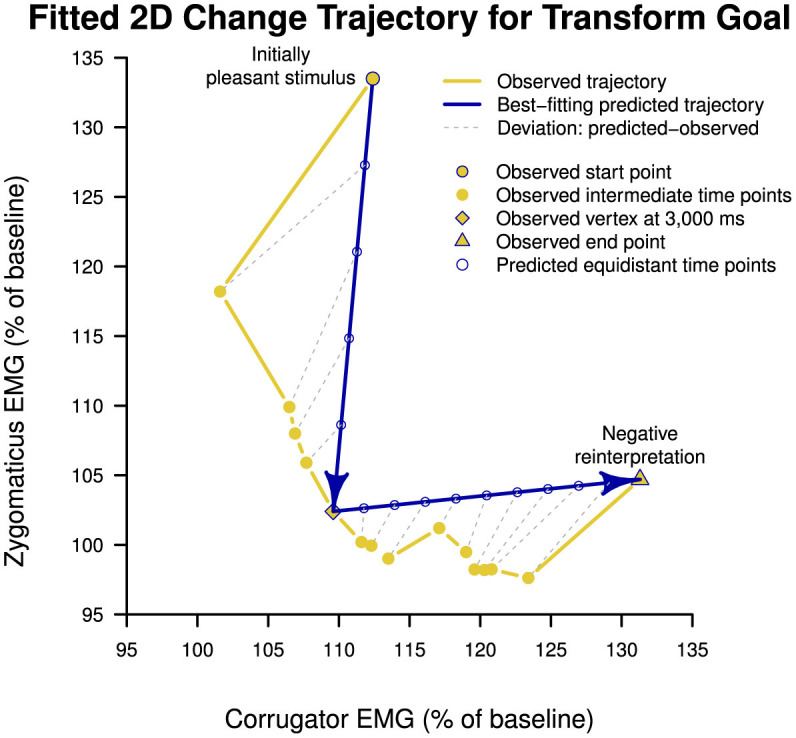
Best-fitting model of temporal trajectory of change brought about by cognitive reappraisal of pleasant stimuli under transform goal condition (i.e., reinterpreting pleasant stimuli with a negative meaning) through two-dimensional affective space spanned by corrugator supercilii and zygomaticus major electromyography reactivity. The best-fitting prediction followed a neutral trajectory as proposed under Hypothesis 3a, describing an L-shape function through affective space. The transition from primarily zygomaticus decrease to corrugator increase occurred at 3,000 ms. EMG, electromyography.

Model fitting of neutral (L-shape, H3a), mixed-emotion (inverse-step-function, H3b), and valence trajectories (diagonal, H3c) to the observed data of the transform goal condition indicated that the neutral trajectory with a vertex at 3,000 ms (time point #6) for changing from the vertical direction (i.e., PA reduction) to the horizontal direction (i.e., NA increase) best fit the data (*MSD* = 55.56). We illustrate the fit of this hypothetical trajectory to the observed data of the transform goal condition in Figure 4 in blue and yellow, respectively. Lowest mean squared deviation for the mixed-emotion trajectory was achieved with a vertex at 1,000 ms (*MSD* = 682.47). The diagonal trajectory resulted in an intermediate deviation (*MSD* = 360.07). We report the full model-fitting results in Table 4. Results of model-fitting analysis suggest that the trajectory of the transform goal through two-dimensional space spanned by corrugator and zygomaticus reactivity was best described by an L-shape trajectory of passing through a neutral emotional state, as hypothesized under H3a.

**Table 4:** Results of model-fitting analysis for neutral, mixed-emotion, and valence trajectory models.

**Fit type**	**Vertex location**	**MSD**
H3a: Neutral trajectory (L-shape)	2	114.33
	3	64.89
	4	60.78
	5	59.44
	6	55.56
	7	61.88
	8	75.69
	9	90.00
	10	135.06
	11	147.13
	12	156.98
	13	177.69
	14	200.14
	15	223.80
H3b: Mixed-emotion trajectory (inverse-step-function)	2	682.47
	3	691.32
	4	708.30
	5	729.21
	6	753.26
	7	779.80
	8	808.27
	9	838.32
	10	869.43
	11	903.96
	12	941.34
	13	980.60
	14	1022.12
	15	1068.87
H3c: Valence trajectory (diagonal)	NA	360.07

Vertex location indicates at which time point the turning point for the L-shape or inverse-step-function were placed for each model fit. For example, a vertex location of 2 for the L-shape indicates that the first arm joined time points 1 to 2 and that the second arm joined time points 2 to 16. Given the presence of a vertex for L-shape and inverse-step-function models, there were 13 points based on which we calculated the sum of squared differences (SSD). Given no vertex for the diagonal model, there were 14 points based on which we calculated the SSD. To make these metrics comparable, we report MSD, rather than SSD. Numbers typeset in bold font indicate minima for L-shape and inverse-step-function models (reported in the main text). MSD, mean squared deviation.

Taken together, results from between-condition and model-fitting analyses indicate that there was simultaneous onset of changes on corrugator and zygomaticus reactivity but that zygomaticus reduction first dominated and—after plateauing—subsequently gave way to more dominant corrugator increase.

## 4 Discussion

**Figure 5:**
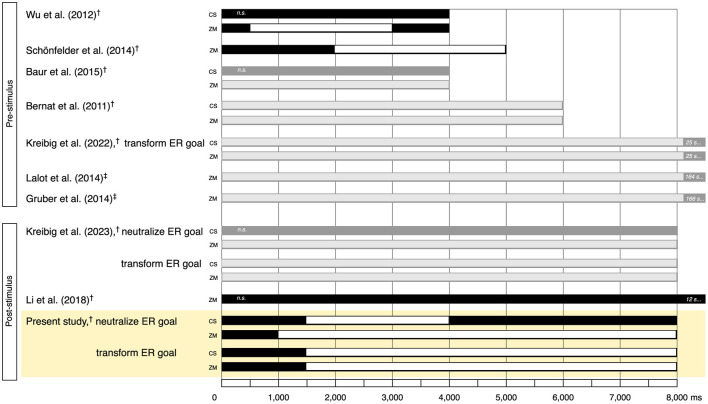
Illustration of onset and duration of effects of down-regulating emotional responses to pleasant stimuli through cognitive reappraisal (CR) as indexed by potential increase of corrugator supercilii and reduction of zygomaticus major. **Top** of figure shows effects as observed in prior studies of pre-stimulus delivery of CR instructions. **Bottom** of figure shows effects as observed in prior studies of post-stimulus delivery of CR instructions. Results illustrated in black and white come from time course studies (black: indication of duration of regulation period; white: indication of effect duration). Results illustrated in dark and light gray come from time average studies (dark gray, indication of duration of regulation period; light gray: indication of significant effect). Yellow highlight marks effects as observed in the present study under neutralize and transform emotion regulation goals. Effects are quantified in comparison to the study's no-regulation condition. ^†^Electromyography (EMG) for quantification of muscle activity. ^‡^Facial Action Coding System (FACS; Ekman and Friesen, 1978) for quantification of muscle activity; Gruber et al. (2014) coded AU12 + AU6 (lip corner puller + cheek raiser); Lalot et al. (2014) coded AU12 (lip corner puller). CS, corrugator supercilii; *n.s*., non-significant; ZM, zygomaticus major.

This study explored how positive ER in terms of reappraisal of pleasant stimuli unfolds over time. We specifically addressed the temporal sequence in which different ER goals in down-regulating positive emotion influence NA and PA response systems. Manipulating neutralize vs. transform ER goals while holding constant other key variables of the ER process (cf. Kreibig et al., 2023) revealed distinct patterns: the neutralize goal effectively reduced PA without (sustained) impact on NA, promoting a neutral emotional state, while the transform goal sequentially reduced PA and then increased NA, leading to a negative emotional state. These time course findings align with previous research on the down-regulation of facial emotional responses to pleasant stimuli, as we summarize in Figure 5. Our results highlight goal-specific regulation of NA and PA, advancing our understanding of how ER goals operate in real-time. These results illuminate the role of individual ER goals for down-regulating positive emotions, offering valuable insights for both affective science and developing targeted ER interventions.

### 4.1 Explaining the sequential pattern of PA–NA change

The finding that, in the down-regulation of positive emotion, like joy or desire, PA transitions to NA through a neutral state (as hypothesized in H3a) prompts questions about the reason for this affective “detour” and its underlying processes. This finding implies a focus on first down-regulating PA rather than directly increasing NA. This suggests that in pursuing a transform goal, the first sub-goal would be to reduce the impact of positive emotion before addressing the second sub-goal of generating negative emotion. Reducing PA might be necessary to resolve the emotionally arousing aspects of the pleasant stimulus, like an image of tempting food, to reduce cognitive and physiological barriers to generating NA. The intermediate neutral emotional state may aid the shift from positive to negative emotions. It could enable individuals to detach from the initial positive emotional reaction, like desire, clearing the emotional canvas and fostering a psychological environment for the subsequent generation of negative reappraisal and the emergence of negative emotions, like disgust.

Changing the cognitive representation of current circumstances, one cognitive reappraisal strategy, involves altering how one mentally represents current situations that might be emotionally evocative in undesired ways to more constructive, adaptive, and emotionally desirable perspectives. This transition may entail initially interpreting the emotional stimulus in a less positive or more emotionally neutral manner, followed by subsequently generating a negative reappraisal.

Opting for a neutral trajectory allows individuals to circumvent potential drawbacks linked with the mixed-emotions trajectory (H3b). While mixed emotions may be beneficial in the longer term (e.g., weeks, months, or years; Adler and Hershfield, 2012; Hershfield et al., 2013; Gabriel et al., 2022), in the moment, mixed emotions extend exposure to emotional arousal, delay attainment of a stable, desired emotional state, create cognitive dissonance arising from conflicting interpretations of stimuli as individuals identify negative aspects while upholding positive appraisals (or vice versa), and may elicit disorientation, distress, and new ER needs due to the incongruity of mixed emotions. These processes may impede the focus on the primary regulatory goal of negative reinterpretation. Rather, with the neutral trajectory, individuals can transition from one univalent state (PA) to another (NA).

While a direct, linear path following the valence dimension (H3c) might be expected intuitively, observation of a neutral trajectory suggests that ER processes are not limited to the shortest geographic distance across the two-dimensional affective space between positive and negative emotional states. Like the mixed-emotions trajectory, the valence trajectory may entail drawbacks, including the increased challenge of regulating two dimensions simultaneously, necessitating the concurrent implementation and monitoring of down-regulating PA and up-regulating NA. This could lead to imprecision in emotional modulation. It could also result in greater emotional discordance, causing psychological discomfort. Hence, instead of a step-wise or cyclical process with small concurrent alterations in PA and NA, complete mitigation of PA seems to be the initial prerequisite for facilitating the generation of NA.

In theoretical terms, the absence of simultaneous changes in NA and PA implies that emotions are not merely bipolar opposites and do not solely change along a single continuum from negative to positive or vice versa. The identification of a neutral trajectory, wherein PA decreases before NA increases, indicates greater emotional complexity, suggesting that emotions can independently change on NA and PA, and regulating one dimension does not have to change the other dimension concurrently. Therefore, traversing a neutral state could be seen as strategic since it might be quicker to introduce a neutral meaning into a pleasant stimulus than to directly infuse it with negativity; it could be simpler to regulate one dimension individually, improving the accuracy of emotional modulation; and it could be the most efficient, adaptive, and emotionally harmonious approach to reaching a transformation goal through reappraisal.

### 4.2 Unexpected corrugator reactivity during the neutralize ER goal

We observed an unexpected rise in corrugator reactivity for the neutralize goal. Compared to the predicted corrugator increase for the transform goal, which started at 1,500 ms, continued to grow throughout the 8,000-ms regulation period, and was of greater magnitude (e.g., a 11.2%-increase when it first became significant and a 23.6%-increase at its highest intensity), the unexpected corrugator response for the neutralize goal showed a later onset at 2,500 ms, peaked at 3,500 ms, discontinued after 4,000 ms, and was of considerably weaker response magnitude (a 6.1%-increase when it first became significant and a 7.2%-increase at its highest intensity). Similarly, compared to the predicted zygomaticus reduction for the neutralize goal, which started at 1,000 ms, continued to decline throughout the regulation period, reached its minimum at 7,000 ms, and was of larger magnitude (e.g., a 12.0%-decrease when it first became significant and a 32.3%-decrease at its lowest intensity), the unexpected corrugator response for the neutralize goal was much more fleeting and feeble.

Given the profound divergence in response characteristics from the other observed (and predicted) responses, we argue that the unexpected corrugator activation of the neutralize goal likely did not reflect an increase in NA activation. It may have rather been due to other processes, such as focused attention or mental effort (Rinn, 1984; Van Boxtel and Jessurun, 1993, cf. Section 4.4), that might have been briefly recruited as the decline in zygomaticus reactivity (PA reduction; range = −13.6 to −2.9 inter-time point differences from 500 to 2,500 ms) decelerated and transitioned into a more stable plateau (range = −3.1 to 2.0 inter-time point differences from 3,000 to 7,500 ms; cf. Section 4.3 on ER phase transitions).

We may speculate that similar processes occurred for the transform goal (PA/zygomaticus reduction: range = −15.3 to −1.9 inter-time point differences from 500 to 2,500 ms; range = −3.5 to 2.2 inter-time point differences from 3,000 to 7,500 ms) but might have been concealed by NA activation as a stronger driver for the rise in corrugator reactivity. Future research should strive to untangle the potential co-occurrence of attention, effort, and affect-related processes on the corrugator response during ER.

### 4.3 Broader implications

The process model of ER (Gross, 1998, 2015) acknowledges that individuals can pursue various regulatory goals. The current work, which studied different ER goals such as neutralizing positive emotions and transforming them into negative ones, aligns with the model's perspective on the array of regulatory goals individuals can pursue. It presents evidence that different goals not only result in varied emotional outcomes but also create distinct temporal patterns of emotional change and selective modulation of NA and PA response systems.

Observing distinct emotional outcomes for each ER goal is also consistent with the process model's (Gross, 2015) claim that the success of regulation efforts relies on their alignment with the regulatory goals. In our study, the neutralize goal primarily decreased PA, whereas the transform goal exhibited alterations in both NA and PA. Reducing PA fulfilled the neutralize goal, but further increasing NA was required to fulfill the transform goal.

Our findings additionally correspond with the process model's (Gross, 1998, 2015) view of ER as a complex phenomenon. Viewing emotions as dynamic processes, this model posits that the impact of ER on emotional reactivity does not occur instantaneously but rather unfolds gradually. Thus, ER may not be a uniform process but may rather express through distinct steps and phases in a temporal sequence. Specifically, the study results revealing a PA–NA sequence in the transform goal support ER as a staged process, where positive emotion reduction precedes negative emotion generation. This implies a fixed order, suggesting that regulation mechanisms shift focus from attenuating positivity to amplifying negativity at distinct stages.

We might interpret the data to suggest a two-step ER process for the transform goal, with the effects of one process appearing earlier than those of the other. Might participants have broken down the transform goal by initially pursuing a “neutralize” goal and then a “negativize” goal? An alternative interpretation of our data suggests an integrative implementation process that first addresses PA and then NA. However, with the current data, we cannot definitively decide between these two possibilities. These alternative interpretations offer more detailed insight into how individuals might regulate their emotions, emphasizing the significance of accounting for time-dependent changes when examining ER processes, and highlighting the necessity for a more nuanced investigation of the mechanisms behind the transform goal.

Indeed, the process model of ER (Gross, 2015) suggests a cyclical progression through various stages of ER, which includes applying the regulation strategy, such as implementation in the form of a tactic, and continuously evaluating the strategy's effectiveness through monitoring to determine whether ER efforts should be withdrawn, continued, or altered. Our results are suggestive of both of these stages occurring, as we may deduce changes in the ER process over time from alterations in the trajectory of the EMG signal. The change trajectory of the neutralize goal, characterized by an initial decrease in PA that eventually stabilized at a low level, may indicate the implementation stage, particularly the initiation of ER efforts. This eventually transitioned into monitoring, aimed at sustaining the neutral interpretation of the initially pleasant stimulus for the duration of the trial.

The change trajectory of the transform goal, which involves both the initiation and maintenance of a decrease in PA similar to the neutralize goal, along with an increase in NA beginning concurrently and becoming predominant at 3,000 ms, may additionally indicate the initiation and implementation of ER efforts aimed at increasing NA. At a broader level, this pattern of initially reducing PA and, upon attaining a neutral emotional state, shifting regulation efforts toward increasing NA, might signify the adjustment, switching, or addition of ER efforts aimed at increasing NA within the monitoring stage. This interpretation implies that the ER process itself may undergo change over time.

These theoretical considerations emphasize the intricate and dynamic nature of ER processes. We trust that these interpretations enhance our comprehension of the mechanisms behind ER in real-time settings and serve as a reminder to researchers of the significance of accounting for the temporal dynamics of ER when designing studies and interpreting results. Future ER research should delve deeper into decoding the processes of emotional change involved in ER (e.g., Chow et al., 2005; Kuppens et al., 2010).

### 4.4 Limitations and future directions

The limitations of this study indicate crucial avenues for future research:

First, given that the sample was limited to young healthy adults, future research should investigate ER goal dynamics in broader participant demographics and explore factors affecting the timing of emotional change.

Second, while this study examined the time course of two regulation goals in reappraising static pleasant pictures eliciting general PA, it is essential to investigate whether these results will generalize to different stimulus modalities, such as dynamic auditory stimuli or film clips, or specific discrete positive emotions like excitement, amusement, contentment, or serenity.

Third, this study concentrated on the time course of ER concerning PA. It is necessary to similarly examine the time course of ER in the context of NA. The central question here is whether there will also be evidence of sequential change in ER (see Kreibig and Gross, 2024, for an examination of this research question).

Fourth, while this study examined the temporal dynamics of explicit, instructed, and externally generated ER goals within the realm of reappraisal, it will be important to examine temporal effects of ER goals in other ER strategies, like distraction or expressive suppression, and under different conditions, such as implicit, spontaneous, or internally generated ER goals (Gyurak et al., 2011). It will be essential to test whether our observation of sequential ER effects will generalize across different strategies and characteristics of ER goals.

Fifth, our decision to use time bins of 500-ms averages was influenced by previous literature and the practicality of conducting repeated tests while ensuring appropriate statistical adjustment. However, like any averaging method, there may be insights missed at higher temporal resolutions. We encourage future studies to investigate higher-resolution temporal averaging or consider analyzing raw data.

Sixth, for this initial exploration of the temporal dynamics of ER goals, we deliberately opted for a straightforward traditional statistical analysis approach. However, more sophisticated nonlinear modeling techniques, such as frequency analysis, growth curve analysis, or dampened oscillator modeling, could unveil additional components, processes, and drivers in the temporal dynamics of ER goals (Chow et al., 2005; Kuppens et al., 2010).

Seventh, this study was limited in terms of the dependent variables used to investigate emotional change processes. We intentionally focused on facial EMG responses, using corrugator supercilii as a well-validated indicator for NA and zygomaticus major for PA (Larsen et al., 2003; Heller et al., 2014). Still, facial EMG responses may also reflect other active purposeful processes requiring focused attention and mental effort (Rinn, 1984; Van Boxtel and Jessurun, 1993, cf. Section 4.2) or be subject to social desirability (Weinberger et al., 1979; Pauls and Stemmler, 2003). However, whereas effects of attention and effort should increase corrugator reactivity during reappraisal independent of stimulus valence, convergent results across the present and companion studies (Kreibig and Gross, 2024) document increased corrugator reactivity for negatively reappraising pleasant stimuli and decreased corrugator reactivity for positively reappraising unpleasant stimuli. Additionally, analysis of potential influences of experimental demand in our prior paper on time-averaged results of the present dataset (Kreibig et al., 2023, Section 4.1.4) indicated no relationship between regulated emotional experience and an individual difference measure of social desirability. Prior research also did not find repressive–defensive coping styles, as derived from social desirability measures, to influence facial EMG responses in affective picture viewing or imagery paradigms (Slomine and Greene, 1993; Houtveen et al., 2001), as used in the present study. This gives us reason to believe that facial EMG responses primarily indexed affect in this experimental context. Nevertheless, future research should explore what distinct cognitive, behavioral, or physiological processes underlie the sequential reduction of PA and increase of NA to further elucidate the change mechanisms of the neutralize and transform goals. Furthermore, researchers may wish to investigate the neurobiological underpinnings of the observed temporal dynamics and whether distinct neural pathways or brain regions are associated with reducing PA as contrasted to enhancing NA in positive ER (cf. McRae et al., 2008; Wager et al., 2008).

Finally, the current findings are derived from participant and trial averages, resulting in a smoothed response curve. However, it remains uncertain whether this average response pattern is also evident at the individual participant or trial level. It will be critical for future research to investigate the presence of variability between individuals and trials within individuals to gain a deeper understanding of the role of individual differences and situational variability in the temporal dynamics of ER goals (e.g., Chow et al., 2005; Kuppens et al., 2010). This research direction may unveil differences in skill levels in implementing ER goals (e.g., Heller et al., 2009), contribute to a better characterization of ER functioning in mental health disorders, and lay the groundwork for more precise interventions in ER.

To sum up, this study showed that the transform goal, aiming to convert a positive emotion into a negative one through reappraisal, created sequential change. Initially, it affected the PA dimension (zygomaticus), followed by the NA dimension (corrugator). Moreover, it showed that the transform goal initially followed a similar trajectory of decreasing PA as the neutralize goal until the effects of increasing NA started to dominate.

## Data availability statement

The datasets presented in this study can be found in online repositories. The names of the repository/repositories and accession number(s) can be found below: all research materials, data, and analysis code will be openly accessible at the OSF repository for transparency and reproducibility: osf.io/7c4zj.

## Ethics statement

The studies involving humans were approved by Stanford University Institutional Review Board, CA, USA (FWA00000935). The studies were conducted in accordance with the local legislation and institutional requirements. The participants provided their written informed consent to participate in this study.

## Author contributions

SK: Conceptualization, Data curation, Formal analysis, Funding acquisition, Investigation, Methodology, Project administration, Validation, Visualization, Writing—original draft, Writing—review & editing, Supervision. JG: Funding acquisition, Resources, Supervision, Writing—review & editing, Conceptualization, Investigation, Methodology.

## Footnotes

^1^Some transform goals may keep emotions within the same area of affective space, such as transforming one discrete high-PA emotion, e.g., joy, into another, e.g., desire.

^2^Though ER efforts might stop earlier if the benefit seems sufficient compared to further effort (e.g., Silvestrini and Gendolla, 2009).

^3^Withdrawal of ER efforts could lead to a rebound of the initial emotional response.

^4^Two participants had α scores of less than 0.60 and five participants less than 0.70, while the large majority of participants (*N* = 149) had α scores larger than 0.70, indicating acceptable reliability and stability of data points throughout the baseline period.

^5^One participant had an α score of 0.41, while the remainder of participants (*N* = 155) had α scores larger than 0.80, indicating acceptable reliability and stability of data points throughout the regulation period.

^6^One participant had a negative α score and eight participants had α scores between 0.60 and 0.70, while the large majority of participants (*N* = 147) had α scores larger than 0.70, indicating acceptable reliability and stability of data points throughout the regulation period.
